# Phase-Sensitive Inversion-Recovery MRI Improves Longitudinal Cortical Lesion Detection in Progressive MS

**DOI:** 10.1371/journal.pone.0152180

**Published:** 2016-03-22

**Authors:** Asaff Harel, Antonia Ceccarelli, Colleen Farrell, Michelle Fabian, Jonathan Howard, Claire Riley, Aaron Miller, Fred Lublin, Matilde Inglese

**Affiliations:** 1 Department of Neurology, Mount Sinai Hospital, New York, New York, United States of America; 2 Department of Radiology, Mount Sinai Hospital, New York, New York, United States of America; 3 Department of Neuroscience, Icahn School of Medicine at Mount Sinai, New York, New York, United States of America; 4 Corinne Goldsmith Dickinson Center for MS, Icahn School of Medicine at Mount Sinai, New York, New York, United States of America; 5 Department of Neurology, New York University, Langone Medical Center, New York, New York, United States of America; 6 Department of Neurology, The Neurological Institute of New York, Columbia University, New York, New York, United States of America; Medical University Vienna, Center for Brain Research, AUSTRIA

## Abstract

Previous studies comparing phase sensitive inversion recovery (PSIR) to double inversion recovery (DIR) have demonstrated that use of PSIR improves cross-sectional *in vivo* detection of cortical lesions (CL) in multiple sclerosis. We studied the utility of PSIR in detection/characterization of accrual of CL over time in a 1-year longitudinal study in primary progressive multiple sclerosis (PPMS) compared to DIR. PSIR and DIR images were acquired with 3T magnetic resonance imaging (MRI) in 25 patients with PPMS and 19 healthy controls at baseline, and after 1 year in 20 patients with PPMS. CL were classified as intracortical, leucocortical or juxtacortical. Lesion counts and volumes were calculated for both time points from both sequences and compared. Correlations with measures of physical and cognitive disability were determined as well as new CL counts and volumes. Compared to DIR, PSIR led to detection of a higher number of CL involving a larger proportion of patients with PPMS both cross-sectionally (p = 0.006, 88%) and longitudinally (p = 0.007, 95%), and led to the reclassification of a third of CL seen on DIR at each time point. Interestingly, PSIR was more sensitive to new CL accumulation over time compared to DIR. PSIR is a promising technique to monitor cortical damage and disease progression in patients with PPMS over a short-term follow-up.

## Introduction

Neuropathological and magnetic resonance imaging (MRI) studies with double inversion recovery (DIR) have shown that cortical lesions (CL) are frequent in multiple sclerosis (MS), [1–17] and play a role in both physical^5^ and cognitive disability, [6–10]. However, DIR has shown low sensitivity especially in detecting subpial lesions [2, 11] and in providing an accurate CL classification [12]. More recently, phase sensitive inversion recovery (PSIR), a T1-weighted sequence with higher signal-to-noise ratio, intensity and gray-white matter contrast has been shown to improve CL detection and classification over DIR cross-sectionally in MS patients. [12–14]

Although cortical demyelination is a predominant feature of PPMS, few MRI studies have investigated the clinical relevance of CL in these patients, [12, 15–18] Studies using DIR [15, 16] have shown that CL in primary progressive multiple sclerosis (PPMS) are similar in frequency, distribution and count to those of relapsing remitting forms, accumulate over time, and correlate with disease progression. Similar results have been found in a recent study using ultra-high-field MRI [17]. However, another study using both DIR and PSIR found a lower CL count in PPMS patients [12]. No studies have evaluated longitudinal CL accrual using PSIR compared to DIR in PPMS.

Thus, the aims of our study were to investigate the utility of PSIR in detecting and characterizing CL evolution in PPMS over a 1-year follow-up and to assess the clinical impact of short-term CL accrual compared to DIR.

## Materials and Methods

### Subjects

The study was approved by the Institutional Review Board of the Icahn School of Medicine at Mount Sinai and written informed consent was obtained from all participants prior to enrollment. Twenty-five patients with MS were prospectively enrolled in the study with the following inclusion criteria: 1) established diagnosis of PPMS according to revised McDonald criteria [19, 20]; 2) age between 25–65 years; 3) Expanded Disability Status Scale (EDSS) score [21] lower than 6.5; 4) if on disease modifying treatment (DMT) they had to have started six months prior to enrollment; 5) no other major medical, neurologic or neuropsychiatric disorder or history of substance abuse; 6) no contraindication to MRI. Twelve PPMS patients (48%) were on DMT (eight with Glatiramer acetate, two with Interferon β1a, and two with Fingolimod). Each patient underwent neurologic examination and brain MRI at baseline. The neurological examination included the EDSS [21], the timed 25-foot walk (T25FW) test, the Nine-Hole Peg Test (NHPT), and the symbol digit modality test (SDMT) [22]. After 1 year, 20 out of 25(80%) PPMS patients underwent a follow-up brain MRI (mean = 1.2 years; SD = ±0.2) and clinical assessment. Dropouts were due to relocation from the study area and patients’ reluctance to continue in the study. Nineteen volunteers (11 women and 8 men; mean age = 52.2 ±9.4 years) with no neurological conditions served as healthy controls (HC) for the baseline MRI assessment.

Baseline and follow-up demographic and clinical data are summarized in Table 1.

**Table 1:** Baseline and follow-up demographics and clinical characteristics of patients with primary progressive multiple sclerosis.

	PPMS patients at baseline (n = 25)	PPMS patients at 1 year follow-up (n = 20)	*p
Female/male	13/12	12/8	-
Age (years)	50.6± 10.4	50.4 ± 11.1	-
Disease duration (years)	8.7± 4.8	9.1 ± 5.0	-
EDSS score median (range)	4 (1.5–6)	4 (2–6)	n.s.
25FWTsec	7.4 ± 2.2	7.1 ± 2.1	n.s.
NHPTsec	31.1 ± 11.7	33.7 ±18.3	n.s.
SDMT raw score	36 ± 14.3	36.5 ± 13.6	n.s.

Values in table are: mean +/- standard deviation; PPMS = primary progressive multiple sclerosis; EDSS = Expanded Disability Status Scale; 25FWT = 25 Feet Walk Test; NHPT = Nine Hole Peg Test; SDMT = Symbol Digit Modality Test

*Wilcoxon signed-rank test p<0.05 in 20 patients with PPMS between baseline and follow-up.

At follow-up, patients were considered clinically worsened if they had an EDSS score increase >1.0 when baseline EDSS was ≤6.0, or an EDSS score increase >0.5 when baseline EDSS was ≥6.0. A worsening of 20% was considered significant for the other clinical measures.

### Image Acquisition

MRI was performed at 3T (Philips Achieva, The Netherlands) using an 8-channel SENSE receive-only head coil with the same protocol for both baseline and follow-up, and patients were repositioned according to established guidelines [23]:

a) Axial dual echo (DE) turbo spin echo (TSE) (TR/TE1/TE2 = 2500/10/80 msec, FoV = 230 mm^2^, matrix size = 512x512), and b) Axial fluid-attenuated inversion recovery (FLAIR) (TR/TE/TI = 9500/120/2400ms, FoV = 256 mm^2^, matrix = 256x256) were acquired with 46 contiguous 3mm-thick slices;

c) Sagittal 3D T1-weighted turbo field echo (TFE): TR/TE/TI = 7.5/3.5/900ms, voxel size = 1mm^3^.

d) Axial DIR (TR/TE/TI_1/2_ = 11000/25/3400_/_325ms) and e) Axial PSIR (TR/TE/TI = 4500/8/400ms) were acquired with FOV = 250mm^2^, matrix size = 256x256, 46 contiguous 3mm-thick slices, reconstructed in-plane resolution = 0.5 x 0.5 x3 mm^3^, acquisition time = 6.36 minutes.

### Image Analysis

All images were analyzed off-line and inspected for presence of major artifacts.

#### Cortical lesion count and volumes

Both at baseline and at follow-up, CL were identified by a single observer blinded to clinical information and subject’s identity, under the supervision of a senior investigator, using T2, T1 and FLAIR images as reference when needed (Jim version 6, Xinapse Systems149 England). PSIR lesions were identified separately from DIR lesions, and the results were subsequently compared. CL were counted according to published guidelines [12, 24] and were differentiated into two subtypes: intracortical lesions (IC) and leucocortical lesions (LC). IC lesions were defined as lesions that were confined to the cortex, while lesions that involved both cortex and juxtacortical white matter (WM) were identified as LC. Juxtacortical lesions (JC) lesions were differentiated from LC lesions based on preservation of normal cortical contour and absence of cortical involvement. Particular care was paid to exclude cortical vessels, Virchow Robin spaces and artifacts [12, 13, 24, 25]. CL were then segmented on both DIR and PSIR using a semi-automated edge-finding tool based on local thresholding technique. Total CL, IC, LC and JC volumes and counts were measured.

#### Reclassification of cortical lesions

Both at baseline and at follow-up, CL that were identified on DIR were compared slice by slice with those identified on PSIR in order to obtain a corresponding classification as previously described [12, 24].

#### Accrual of new cortical lesions

For both DIR and PSIR, counting of new CL was performed slice by slice. A reclassification analysis for the new CL was also performed comparing new DIR lesions with new PSIR lesions as described above.

#### Reproducibility

Intra-rater reproducibility was calculated using the intraclass correlation coefficient (ICC) on baseline data. Using DIR, the ICC was 0.98, 0.99 and 0.96 respectively for TCL, IC and LC. Using PSIR, the ICC was 0.98, 0.98 and 0.97 respectively for TCL, IC and LC.

#### Brain and lesion volumes

Both at baseline and at follow-up, T2 and T1 lesion (T2LV and T1LV), normalized brain (NBV), gray matter (NGMV), and white matter (NWMV) volumes were measured as previously published [26, 27].

Part of the results related to PSIR evaluation of GM lesions at baseline has been published [28].

### Statistical analysis

All statistical analyses were performed using Statistical Package for the Social Sciences (SPSS, Version 20.0, Chicago, Illinois). At baseline, PPMS and HC were compared in terms of age and sex using respectively a Mann Whitney test and the Fisher exact test. Wilcoxon signed-rank test was used to compare PSIR and DIR lesion counts and volumes in the MS cohort at both time points, and to assess paired differences in clinical and MRI variables between baseline and follow-up. Spearman’s rank correlation coefficients were used to determine the associations between CL counts and volumes at both time points with baseline and follow-up demographic, clinical and other MRI variables. Partial correlations were used for taking into account age and sex. All p values are reported as two-sided significance levels and considered statistically significant when p≤0.05.

## Results

No significant group differences were observed when comparing HC and patients with PPMS with regard to age (*p* = 0.6) and sex (*p* = 0.5). No statistically significant differences were found in clinical measures between baseline and follow-up in patients with PPMS (Table 1). EDSS worsened in 2 patients at follow-up while a 20% worsening of NHPT, 25FWT or SDMT was found in 5 PPMS patients.

**Brain and lesion volumes.** A total of fourteen non-specific, age-related T2 hyperintense WM lesions were found in nine HC. In PPMS patients, baseline T2LV and T1LV were: mean±SD = 5.8±7.5 cm^3^and mean±SD = 3.1±4.9 cm^3^, respectively. As expected, HC showed higher NGMV (mean±SD = 758.2±39.8 cm^3^), NWMV (mean ±SD = 674.9±37.1 cm^3^), and NBV (mean ±SD = 1433.1±54.96 cm^3^) compared to patients (NGMV mean ±SD = 724.9±39.8 cm^3^; NWMV mean ±SD = 648.5±46.01 cm^3^; NBV mean ±SD = 1373.4±62.7 cm^3^) (p = 0.014; p = 0.048; p = 0.003, respectively).

**Fig 1:**
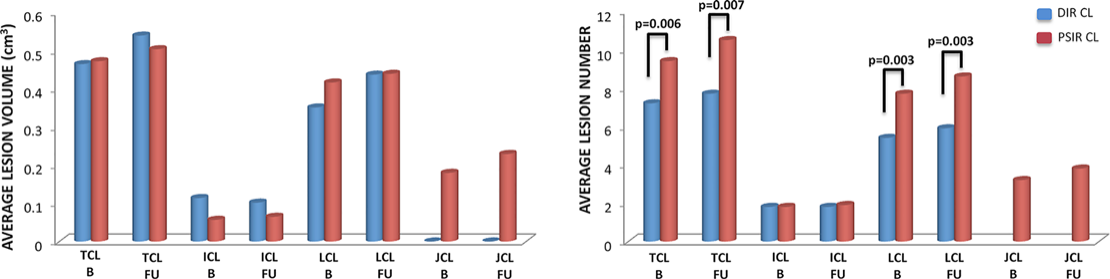
Baseline and follow-up DIR and PSIR cortical lesion volumes and numbers. At baseline (B) no statistically significant differences were detected between DIR and PSIR cortical lesion volumes while differences were found in cortical lesion numbers. PSIR detected a higher number of cortical lesions and leucocortical lesions compared to DIR. Similarly; at follow-up (FU) no statistically significant differences were detected between DIR and PSIR cortical lesion volumes, while differences were found in cortical lesion counts. PSIR detected a higher number of cortical lesions and leucocortical lesions compared to DIR. Furthermore, PSIR at both time points detected juxtacortical lesions.

In the 20 PPMS patients who underwent follow-up MRI, there were no statistically significant differences in T2LV between baseline and follow-up (baseline mean ±SD = 6.4±8.0cm^3^; follow-up mean ±SD = 6.9±8.8 cm^3^), while T1LV was significantly higher at follow-up (baseline mean±SD = 3.5±5.3 cm^3^; follow-up mean ±SD = 4.2±6.6 cm^3^, p = 0.006). No statistically significant differences were found between baseline [NGMV (mean±SD = 726.2±43.0 cm^3^); NWMV (mean±SD = 645.1±44.4 cm^3^); NBV (mean±SD = 1371.3±66.0 cm^3^)] and follow-up brain volumes [NGMV (mean ±SD = 725.8±42.7 cm^3^); NWMV (mean ±SD = 641.8±41.7 cm^3^); NBV (mean ±SD = 1367.4±63.3 cm^3^)]. Baseline and follow-up CL volumes detected by DIR and PSIR in PPMS patients are summarized in Table 2 and Fig 1. No statistically significant differences were found between DIR and PSIR volumes at baseline and at follow-up.

**Table 2:** Baseline and follow-up cortical lesion volumes and counts in patients with primary progressive multiple sclerosis.

	Baseline (n = 25)	*p	Follow-up (n = 20)	**p	***p
**CL number**					
DIRTotal CL	7.2 ± 6.8 (179)	0.006	7.7 ± 6.7 (153)	0.007	n.s.
PSIR Total CL	9.4 ± 9.1 (236)		10.5 ± 9.5 (210)		0.008
DIR IC	1.8 ± 2.0 (45)	n.s.	1.8 ± 2.1 (35)	n.s.	n.s.
PSIR IC	1.8 ± 1.96 (44)		1.9 ± 2 (38)		n.s.
DIR LC	5.4 ± 5.2 (134)	0.003	5.9 ± 5.3 (118)	0.003	n.s.
PSIR LC	7.7 ± 7.7 (192)		8.6 ± 8.1 (172)		0.014
PSIR JC	3.2 ± 3.97 (79)	-	3.8 ± 4.3 (75)	-	n.s.
**CL volume**					
DIRTotal CLV	0.46 ± 0.48	n.s.	0.54 ± 0.52	n.s.	n.s.
PSIR Total CLV	0.47 ± 0.45		0.50 ± 0.48		n.s.
DIRICV	0.11 ± 0.14	n.s.	0.10 ± 0.13	n.s.	0.036
PSIR ICV	0.06 ± 0.06		0.07 ± 0.08		n.s.
DIRLCV	0.35 ± 0.39	n.s.	0.44 ± 0.46	n.s.	n.s.
PSIR LCV	0.42 ± 0.43		0.44 ± 0.47		n.s.
PSIR JCV	0.18 ± 0.20	-	0.23 ± 0.20	-	n.s.

Values in table are: mean +/- standard deviation; lesion volumes are in cm3. in () = total number of lesions detected; DIR = double inversion recovery; PSIR = phase sensitive inversion recovery; CL = cortical lesions; IC = intracortical lesions; LC = leucocortical lesions; JC = juxtacortical lesions; CLV = cortical lesion volume; ICV = intracortical lesion volume; LCV = leucocortical lesion volume; JCV = juxtacortical lesion volume.

Wilcoxon signed-rank test p<0.05 was used to compare PSIR and DIR lesion counts and volumes in the MS cohort at baseline* and at follow-up**. ***Wilcoxon signed-rank test p<0.05 in 20 PPMS patients between baseline and follow-up.

### Baseline cortical lesion count and classification

#### Healthy controls

A total of 2 IC lesions on DIR and a total of 2 JC lesions on PSIR were identified in 11% (2 out of 19) of HC. None of the 2 IC lesions seen on DIR were visualized on PSIR.

#### PPMS patients

CL were identified in 84% (21 out of 25) of patients on DIR and in 88% (22 out of 25) of patients on PSIR. Compared to DIR, a higher number of total CL (p = 0.006) and LC (p = 0.003) lesions was detected on PSIR. In addition, 79 JC lesions were detected on PSIR (Table 2). Out of all lesions identified on DIR [(IC+LC) = 179)], 75% were LC and 25% were IC. Of all lesions identified on PSIR [(IC+LC+JC) = 315], 61% were LC, 14% IC and 25% JC.

#### Reclassification of cortical lesions

Overall, 65.4% of CL seen on DIR were confirmed, 30.2% were reclassified as different CL, and 4.5% were not visualized on PSIR. Of LC lesions on DIR, 73.1% were confirmed as LC, 21% reclassified as JC, 3.7% as IC, and 2.2% were not visualized on PSIR. Of IC lesions on DIR, 42.2% were confirmed as IC, 46.7% reclassified as LC, and 11% were not visualized on PSIR. Overall, 27% of LC lesions and 57.7% of IC lesions were reclassified.

### Follow-up cortical lesion count and classification

#### PPMS patients

CL were identified in 90% (18 out of 20patients) on DIR and 95% (19 out of 20) of patients on PSIR. Compared to DIR, a higher number of total CL and LC lesions was detected on PSIR. In addition, 75 JC lesions were detected on PSIR (Fig 1 and Table 2). Out of all DIR lesions [(IC+LC) = 150], 77% were LC and 23% were IC. Out of all PSIR lesions [(IC+LC+JC) = 285], 60% were LC,13% were IC, and 27% were JC.

#### Reclassification of cortical lesions

Overall, of the CL seen on DIR, 63% were confirmed, 34% were reclassified, and 3.3% were not visualized on PSIR.

Of LC lesions seen on DIR, 69.5% were confirmed as LC, 24.6% reclassified as JC, 4.2% as IC, and 1.7% were not visualized on PSIR. Of IC lesions seen on DIR, 40% were confirmed as IC, 51.4% as LC, 8.6% were not visualized, and none were identified as JC on PSIR. Overall, 30.5% of LC lesions and 60% of IC lesions were reclassified on PSIR.

### Accrual of new cortical lesions

#### PPMS patients

New CL were identified in 15% (3 out of 20) of patients on DIR, and 35% (7 out of 20) on PSIR. On DIR, these lesions were all LC. On PSIR, 6 were LC and 1 was IC. A significant increase in the number of total CL and LC lesions was seen on PSIR (see Table 2 and Fig 1) over time.

#### Reclassification of new cortical lesions

**Fig 2:**
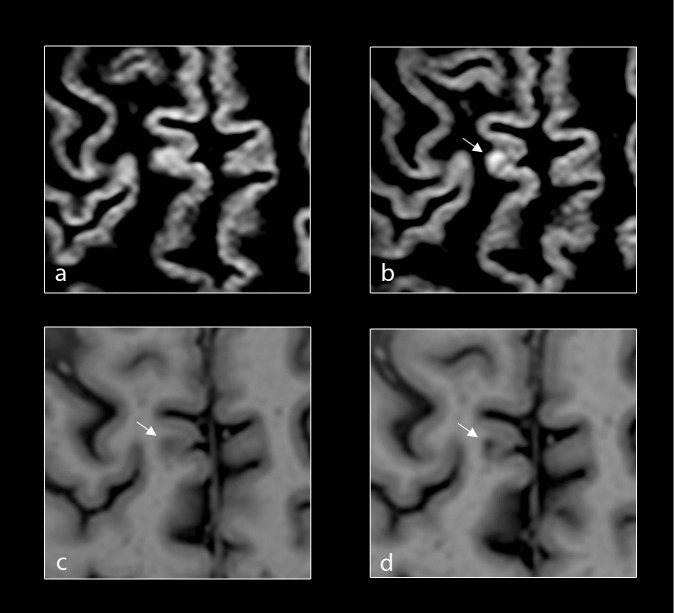
Reclassification of new cortical lesions. Baseline and follow up axial DIR (a, b) of the brain of a patient with primary progressive multiple sclerosis (PPMS) demonstrating a new focal lesion in the cortical grey matter (white-arrows) at follow-up. Corresponding baseline and follow-up axial PSIR (c, d) of the same patient with PPMS demonstrating focal lesions in the cortical grey matter (white-arrows) at both baseline and follow-up.

Overall, of the 3 new CL on DIR, 66% were confirmed and 33% were reclassified on PSIR. One was JC on PSIR and 2 LC were confirmed as LC but already present on PSIR at baseline, and thus were not considered new on PSIR (Fig 2).

**Fig 3:**
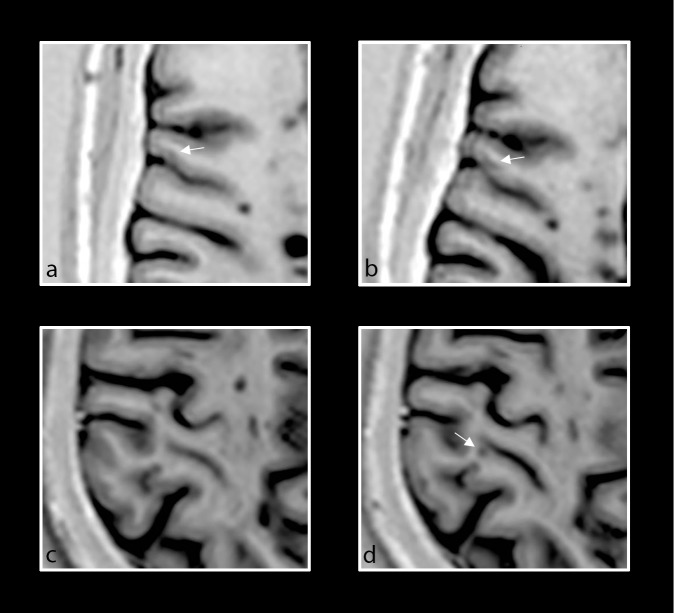
Retrospective analysis of new PSIR cortical lesions. Baseline and follow-up axial PSIR images of different patients with primary progressive MS demonstrating focal lesions in the cortical grey matter. Intracortical lesion that was too small to be counted at baseline (a, white arrow), but that enlarged and was counted on follow-up scan (b, white arrow). New LC lesion (d, white arrow) noted at follow-up but not at baseline (c).

Furthermore, on careful retrospective analysis of the 7 new CL on PSIR, one IC lesion and 4 of the LC lesions appeared to have been present on baseline PSIR but had not met criteria to be marked as lesions per prior established guidelines [24], (Fig 3). The average volume of these 5 lesions was 0.00661±0.0016 cm^3^ at baseline and 0.019±0.0049 cm^3^ at follow-up, translating to an average increase of 188%. 2 LC lesions were completely new on PSIR.

### Correlations between CL volumes and counts and MRI and clinical measures

Overall no clinical changes were observed at 1-year follow-up as stated in the paragraph above, thus correlations with clinical measures were performed only at each time point.

#### Baseline correlations

Correlations were found between CL and raw SDMT and NHPT scores (Table 3). Once age and gender were taken into account, correlations remained between NHPT with PSIR ICV (r = 0.69; p = 0.0001) and PSIR IC count (r = 0.62; p = 0.002). No significant correlations were found with demographic variables. As expected based on evidence from previously published studies, several correlations were found with other MRI variables (data not shown).

**Table 3:** Significant baseline and follow-up correlations between CL counts and volumes and clinical variables (without correction for age and gender).

	Baseline				Follow-up							
	PPMS (n = 25)				PPMS (n = 20)							
	SDMT		NHPT		EDSS		SDMT		NHPT		25FWT	
**CL number**	r	p	r	p	r	p	r	p	*r*	*p*	*r*	*p*
DIR Total CL	-0.50	0.02	0.42	0.04	n.s.	n.s.	-0.62	0.003	0.51	0.02	0.47	0.04
PSIR Total CL	-0.52	0.01	n.s.	n.s.	n.s.	n.s.	-0.69	0.001	0.47	0.04	0.45	0.05
DIR IC	-0.47	0.02	0.41	0.04	n.s.	n.s.	-0.56	0.01	0.54	0.01	n.s.	n.s.
PSIR IC	-0.59	0.003	0.60	0.002	n.s.	n.s.	-0.82	<0.0001	0.71	<0.0001	n.s.	n.s.
DIR LC	-0.50	0.02	n.s.	n.s.	0.46	0.04	-0.55	0.01	0.46	0.04	0.52	0.02
PSIR LC	-0.50	0.02	n.s.	n.s.	n.s.	n.s.	-0.61	0.005	n.s.	n.s.	0.48	0.03
**CL volume**												
DIR Total CLV	-0.44	0.03	n.s.	n.s.	0.50	0.03	-0.53	0.02	n.s.	n.s.	0.50	0.03
PSIR Total CLV	-0.50	0.01	n.s.	n.s.	n.s.	n.s.	-0.66	0.002	n.s.	n.s.	0.50	0.04
DIR ICV	n.s.	n.s.	n.s.	n.s.	n.s.	n.s.	-0.52	0.02	n.s.	n.s.	n.s.	n.s.
PSIR ICV	-0.54	0.01	0.64	0.01	n.s.	n.s.	-0.75	<0.0001	0.78	<0.0001	n.s.	n.s.
DIR LCV	-0.48	0.02	n.s.	n.s.	0.55	0.012	-0.49	0.03	n.s.	n.s.	0.52	0.02
PSIR LCV	-0.47	0.02	n.s.	n.s.	0.52	0.02	-0.55	0.01	n.s.	n.s.	0.52	0.02

r and p derived from Spearman rank correlations coefficient, DIR = double inversion recovery; PSIR = phase sensitive inversion recovery; PPMS = primary progressive multiple sclerosis; CL = cortical lesions; IC = intracortical lesions; LC = leucocortical lesions; JC = juxtacortical lesions; CLV = cortical lesion volume; ICV = intracortical lesion volume; LCV = leucocortical lesion volume; JCV = juxtacortical lesion volume. EDSS = Expanded Disability Status Scale; SDMT = Symbol Digit Modality Test; NHPT = Nine Hole Peg Test; 25FWT = 25 Feet Walk Test.

#### Follow-up correlations

Correlations with clinical variables (Table 3) were found. When age and gender were taken into account, correlations were found between SDMT score and PSIR IC number (r = -0.53, p = 0.02) and volume (r = -0.62, p = 0.004), and between 25TFW and DIR LCV (r = 0.49,p = 0.04), DIR CL number (r = 0.58, p = 0.01), DIR LC number (r = 0.59, p = 0.01), PSIR CL number (r = 0.55, p = 0.02), and PSIR LC number (r = 0.56, p = 0.02). No significant correlations were found with demographic variables.

As expected from previously published studies, several correlations were found with other MRI variables (data not shown).

## Discussion

Our study confirmed that the use of PSIR over DIR leads to better detection and a refined classification of CL [12, 14]. Specifically, the use of PSIR confirms that the majority of PPMS patients exhibit CL. No CL were detected in HC on PSIR, reinforcing previous observations [12] and further suggesting that CL might be useful in increasing the accuracy of MRI diagnostic criteria for MS. Moreover, PSIR detected a greater number of CL and LC and allowed separation of LC and JC lesions. Finally, use of PSIR led to the reclassification of around a third of all CL seen on DIR. Specifically, more than half of the IC lesions and almost a fourth of the LC lesions seen on DIR were reclassified on PSIR. Not surprisingly, the majority of reclassified LC lesions were identified as JC on PSIR, and the majority of reclassified IC lesions were identified as LC on PSIR. Thus, as suggested by previous studies, [12, 14] DIR imaging is not as accurate with regards to CL classification, and this can be improved with the use of PSIR. However, the improvement in CL detection and reclassificationon PSIR was lower than in previous studies [12,14]. Several factors may have contributed to the different results, including the lower spatial resolution of our PSIR sequence, the different head coil design, and, likely, the smaller sample size and different cohort of patients.

Nevertheless, this is the first study to assess accrual of new CL over time using PSIR compared to DIR in patients with PPMS. We showed that more than a third of patients with PPMS exhibited one new CL on PSIR at follow-up. Only a few new CL were detected on DIR, in contrast with a previous PPMS study in which a large accumulation of new IC lesions was detected on DIR over 2 years [16]. This may be accounted for by differences in follow-up length (1 year vs. 2 year), sample size, clinical characteristics, and MRI sequences characteristics and field strength. Of the 3 new LC lesions detected on DIR, two had been visualized on baseline PSIR, reinforcing the idea that PSIR has a higher sensitivity for CL compared to DIR. As previously described [29], MRI visualization of CL depends mostly on CL size ("tip of the iceberg" effect), and intensity and sensitivity of the sequence. Thus, a larger lesion size would be necessary for detection on DIR, and this could lead to a delay in identification of CL with this sequence. Of the 7 new CL identified on PSIR, a retrospective analysis showed that the IC and four of the LC lesions were already present at baseline but they had not met inclusion criteria to be considered as lesions at the time, and got larger in the follow-up analysis (Fig 3). Taken together these observations could have important implications when confirmed in larger studies. On one hand they can support the use of less stringent criteria for identification of CL when a high resolution PSIR is used, and on the other hand they suggest that accumulation of cortical damage over time is still present and continuous in the progressive phases of the disease, even over a short time interval. Our study depicts enlargement of LC and IC lesions, as well as development of new LC lesions. Although the hypothesis that new LC lesions could be formed by enlarging IC lesions [12] cannot be ruled out given our small sample size, larger studies are warranted.

In line with previous DIR studies, our study shows that CL impact physical and cognitive disability [7–10] in patients with MS. Finally, while there were changes in MRI measures of damage (T1LV increase and new CL accumulation) at 1y follow-up, not surprisingly only a few patients experienced changes in clinical variables over this short term interval, thus limiting our ability to determine predictors of clinical outcomes [30].

Our study has several limitations. First of all, the lower spatial resolution of PSIR compared to two previous studies [12, 14] has likely limited our findings, not only in decreasing our ability to reclassify CL but also in assessing the shape of CL as previously shown. It would be of interest in future studies to define the most characteristic CL shape in PPMS. Second, larger studies are needed to confirm our findings because of our small sample size. Furthermore, we were not able to identify subpial lesions. Subpial lesions have proved hard to detect even with all the MRI sequences available^11^ including high resolution PSIR [12, 14].

## Conclusions

Our study confirms that PSIR allows for better detection and improved classification of CL as compared to DIR at both baseline and short-term follow-up, showing ability to monitor cortical damage and disease progression in patients with PPMS over a short-term follow-up.
